# Open vs. robot-assisted preperitoneal inguinal hernia repair. Are they truly clinically different?

**DOI:** 10.1007/s10029-024-03050-8

**Published:** 2024-05-04

**Authors:** V. Rodrigues-Gonçalves, M. Verdaguer-Tremolosa, P. Martínez-López, N. Fernandes, R. Bel, M. López-Cano

**Affiliations:** Present Address: General Surgery Department, Abdominal Wall Surgery Unit, Hospital Universitari Vall d’Hebron, Universitat Autònoma de Barcelona, Paseo Vall d`Hebron 119-129, 08035 Barcelona, Spain

**Keywords:** Inguinal hernia repair, Open preperitoneal inguinal hernia repair, Posterior mesh inguinal hernia repair, Robotic inguinal hernia repair

## Abstract

**Introduction:**

Inguinal hernia repair lacks a standard repair technique, with laparo-endoscopic and open preperitoneal methods showing similar outcomes. Despite higher costs, the popularity of robotic surgery is on the rise, driven by technological advantages. Controversies persist in comparing open repair techniques with the robotic approach, given contradictory results. The objective of this study was to compare postoperative outcomes, including complications, chronic pain, and recurrence, between open and robotic-assisted preperitoneal inguinal hernia repair.

**Methods:**

This single-center retrospective study encompassed patients undergoing elective inguinal hernia repair in a specialized unit, employing both open preperitoneal and robotic-assisted laparoscopic approaches from September 2018 to May 2023. Comparative analysis of short- and long-term outcomes between these techniques was conducted. Additionally, multivariate logistic regression was employed to explore predictors of postoperative complications.

**Results:**

A total of 308 patients met the inclusion criteria. 198 (64%) patients underwent surgery using an open preperitoneal approach and 110 (36%) using robot-assisted laparoscopy. Patients in the robot-assisted group were younger (P = 0.006) and had fewer comorbidities (P < 0.001). There were no differences between the groups in terms of postoperative complications (P = 0.133), chronic pain (P = 0.463) or recurrence (P = 0.192). Multivariate analysis identified ASA ≥ III (OR, 1.763; 95%CI, 1.068–3.994; P = 0.027) and inguinoscrotal hernias (OR, 2.371, 95%CI, 1.407–3.944; P = 0.001) as risk factors of postoperative complications.

**Conclusions:**

Both open preperitoneal and robotic-assisted laparoscopic approaches show similar outcomes for complications, chronic pain, and recurrence when performed by experienced surgeons. The open preperitoneal approach, with its quicker operative time, may be advantageous for high-comorbidity cases. Treatment choice should consider patient factors, surgeon experience, and healthcare resources.

## Introduction

It is estimated that more than 20 million hernias are repaired worldwide each year [1]. However, there is no standard repair technique for all inguinal hernias [2]. Anterior approaches involving mesh placement (e.g., Lichtenstein) and laparo-endoscopic repair have been the most commonly evaluated methods in the literature [2]. According to clinical guidelines, when performed by an experienced surgeon and compared to anterior approaches involving mesh placement, laparo-endoscopic techniques are associated with faster recovery times and a lower risk of chronic pain [2]. Although data for open preperitoneal techniques are limited, comparable results to those of laparo-endoscopic techniques have been reported in terms of the risk of chronic pain, complications, and recurrence [3].

In the last two decades, with the advent of robotic surgery, an increase in the use of minimally invasive surgery in inguinal hernia repair has been reported, especially in the United States [4]. Studies comparing the conventional laparoscopic approach with the robotic approach show that the robotic approach is more costly and time consuming, while the results in terms of complication, chronic pain and recurrence rates are similar for both methods [5]. Despite these data, a progressive increase in the use of robotic surgery has been observed, probably related to the advantages of this technology (wrist instruments, tremor filtering, and 3D imaging), which facilitate the execution of the technique and can potentially reduce learning curves [6]. On the other hand, the means by which to compare robotic inguinal hernia repair with open techniques are debated. While some studies have reported lower complication rates with robotic surgery than with open anterior repairs [7], others have reported higher rates of complications, chronic pain, and recurrence in patients receiving robotic-assisted repairs [8]. With respect to the open preperitoneal approach, less seroma formation has been reported than with minimally invasive techniques (including robotic surgery), for which the recurrence rates are equivalent [9]. However, there are very few data comparing the open preperitoneal approach with robot-assisted repair, so no conclusions can be drawn about which of these techniques is superior.

The objective of this study was to compare the postoperative results, in terms of complication, chronic pain and recurrence rates, between the open preperitoneal approach and the minimally invasive robot-assisted approach in the elective repair of inguinal hernia.

## Methods

This was a retrospective cohort study performed at the Abdominal Wall Surgery Unit of the Vall d’Hebron University Hospital between September 2018 and May 2023. All patients who underwent inguinal hernia surgery were identified from a database maintained prospectively in our unit. From this group of patients, those who were operated on by surgeons who had experience in both the open preperitoneal repair technique and robotic surgery were selected for analysis. The present study was conducted in accordance with the Declaration of Helsinki, and the Strengthening the Reporting of Observational Studies in Epidemiology (STROBE) [10] and Reporting of Studies Conducted Using Observational Routinely Collected Health Data (RECORD) [11] requirements for observational studies were applied.

### Patients

The inclusion criteria were as follows: (1) age ≥ 18 years and (2) underwent elective inguinal hernia repair by surgeons experienced in both the open preperitoneal and robotic-assisted repair. The exclusion criteria were as follows: (1) underwent surgery performed by surgeons with experience in only one of the techniques and (2) underwent emergency hernia repair.

Patients were followed up by their surgeons 4 weeks after discharge and 6 months after surgery. For the purpose of this study, telephone interviews were conducted to determine the presence of recurrence and chronic postoperative inguinal pain (CPIP).

### Demographic and clinical variables

Demographic variables (age, sex, and body mass index [BMI]) were collected, as was the American Society of Anesthesiologists (ASA) score and the presence of comorbidities (cardiovascular disease, chronic obstructive pulmonary disease [COPD], chronic nephropathy, liver cirrhosis, diabetes, smoking status, and anticoagulant treatment). The side and type of hernia according to the European Hernia Society (EHS) classification [2] were included in the analysis. Additionally, whether the hernia was bilateral, recurrent, or inguinoscrotal was recorded.

### Operative variables

A total of 3 surgeons performed all the interventions, both by open preperitoneal and robot-assisted laparoscopic techniques. The type of approach was chosen at the discretion of the surgeon based on his or her preference and preoperative characteristics. In general, those patients with high comorbidity in whom it is advisable to avoid pneumoperitoneum and prolonged operating times, as well as those patients with previous abdominal surgeries, were mostly selected for an open preperitoneal approach. Female patients and those with bilateral inguinal hernia were mostly selected for R-TAPP following clinical guidelines that recommend the minimally invasive approach in these patients.

### Open preperitoneal approach

Most repairs were performed under general anesthesia, and less frequently, spinal anesthesia was applied at the discretion of the anesthesiologist. All patients received preoperative antibiotics according to the institution’s protocol.

All hernias were repaired using an open standardized method, which is described as follows:

The open preperitoneal approach consisted of a transverse skin incision two centimeters above the symphysis pubis and two centimeters outside the midline, extending the dissection to the subcutaneous cellular tissue, anterior lamina of the rectus muscle and aponeurosis of the oblique muscles. Subsequently, the transversalis fascia was opened to access the preperitoneal space, identifying the sectioned epigastric vessels as necessary and dissecting the medial and lateral preperitoneal space. By means of medial traction of the rectus muscle and the lower edge of the abdominal wall section, the herniation area was identified. After the hernia content was reduced, the cord elements were dissected, and the potential hernia points in the myopectineal orifice of the prosthesis were reviewed. A polypropylene mesh measuring 15 × 15 cm was used to completely cover the myopectineal orifice. The mesh was anchored to the pectineal ligament with a 2–0 absorbable monofilament stitch. A slit was made in the lateral border of the mesh to accommodate the cord elements. After the mesh was spread, the layers of the abdominal wall were anatomically closed.

### Robotic approach

In our hospital, the robotic abdominal wall surgery program began in September 2018 with the Da Vinci Xi robotic platform (Intuitive Surgical, Inc., Sunnyvale, CA). In accordance with the mandatory training recommendations required by the manufacturer of the robotic system and following the indications recommended by the EHS [12], a training plan was developed for three surgeons from the abdominal wall surgery unit. This training consisted of simulator practice, observation of clinical cases performed by an expert surgeon, and performance of the first cases in the presence of a supervising surgeon. Currently, more than 200 robotic-assisted abdominal wall surgery interventions have been performed in our unit.

All patients in the robot-assisted laparoscopic surgery group underwent surgery via the TAPP approach under general anesthesia. The patient was placed supine in a slight Trendelenburg position. A Veress needle was inserted at the supraumbilical level to create pneumoperitoneum. The camera trocar was placed at the midline approximately 15–20 cm from the pubic symphysis, and two additional ports were placed on both sides 8 cm from the camera trocar and in line with it. An incision was made in the peritoneum below the arcuate line from the umbilical ligament to the anterior–superior iliac spine to access the preperitoneal plane. The preperitoneal space developed medially to the pubic symphysis, and the pectineal ligament was identified, extending the dissection 2 cm below the pubic symphysis. In the lateral compartment, the dissection continued to the anterior–superior iliac spine. All hernia sacs that may have been present in the myopectineal orifice were identified and reduced in size. In all patients, a 12 × 15 cm polypropylene mesh was fixed to the pectineal ligament with absorbable sutures. The peritoneum was closed using absorbable sutures.

Other operative details were recorded, including the operative time (time from the incision to the application of dressing) and the incidence of intraoperative complications.

### Postoperative variables

The postoperative variables for this study were postoperative complications (within 90 days postoperatively), CPIP and hernia recurrence. Postoperative complications were defined as any condition that could prolong the length of hospital stay or impact outcomes and were categorized into hematoma, seroma, and acute urinary retention. Acute urinary retention was defined as a case requiring a Nelaton or Foley catheter insertion for voiding. Complications were classified according to the Clavien‒Dindo (CD) classification [13]. The other postoperative variables collected were length of stay and readmission.

Hernia recurrence was determined by screening the medical records for reports of any intervention for ipsilateral recurrent hernia, either during physical examination by the surgeon or during telephone interview in which the Ventral Hernia Recurrence Inventory (VHRI) [14] was administered. The VHRI is a tool that has been validated for use in both inguinal^14^ and ventral [15] hernia populations to evaluate the presence of recurrence. CPIP was defined as pain that persisted for three months or more following surgery [16]. CPIP was evaluated via telephone interview. Patients were asked to report their pain level on a four-point categorical scale (none, mild, moderate, severe) that was validated in previous studies [17, 18]. Mild pain was defined as occasional discomfort that did not limit daily activity after returning to the prehernia lifestyle and did require analgesics. Moderate pain was defined as pain that interfered with the patient’s return to normal daily activities, and analgesics were rarely needed. Severe pain was defined as pain that frequently incapacitated the patient or interfered with everyday activities, and painkillers were frequently needed. All patients who had any positive response on the VHRI and/or who reported any degree of chronic postoperative pain were strongly recommended to schedule a face-to-face visit for a physical examination. For patients who did not respond to the follow-up telephone interviews, the last in-person postoperative visit was considered the last follow-up date.

### Statistical analysis

Quantitative variables are reported as the means and standard deviations and were analyzed using Student’s t test or the Mann‒Whitney U test when necessary. Qualitative variables are expressed as counts and percentages and were compared using the chi-square test or Fisher’s exact test when necessary. A logistic regression model was used for postoperative complication analysis. The inclusion of the variables in the model was based on their significance in the univariate analysis (P < 0.05) and on clinical consensus. The postoperative complication rates are reported as odds ratios (ORs) with 95% confidence intervals (CIs). A two-tailed P value < 0.05 indicated statistical significance. SPSS (IBMS SPSS Statistics 23) was used for statistical analysis.

## Results

### Patient demographics

A total of 308 patients underwent elective inguinal hernia surgery between September 2018 and May 2023. Of these patients, 198 (64%) underwent an open preperitoneal approach, and 110 (36%) underwent an R-TAPP repair. The patients in the R-TAPP group were younger (*P* = *0.006*) and tended to be female (*P* = *0.01*). The patients who underwent the open preperitoneal approach had more comorbidities (*P* < *0.001*), a higher American Society of Anesthesiologists (ASA) classification (*P* = *0.001*), and more previous abdominal surgeries (*P* = *0.005*). There were no differences between the groups in terms of BMI (Table 1).

**Table 1:** . Patient characteristics of study population

Variables	Total (n = 308)	Open preperitoneal group (n = 198)	R-TAPP group (n = 110)	*P* values
Age (yr)[mean (SD)]	72.2 (12.9)	73.6 (12.3)	69.6 (13.5)	0.006
Sex [n, (%)] Male Female	272 (88) 36 (12)	182 (92) 16 (8)	90 (82) 20 (18)	0.010
BMI (kg/m^2^) [mean (SD)]	25.7 (3.2)	25.6 (3.2)	25.8 (3)	0.405
ASA score I/II [n, (%)] III/IV [n, (%)]	169 (55) 139 (45)	95 (48) 103 (52)	74 (67) 36 (33)	0.001
Previous abdominal surgery [n, (%)]	122 (40)	90 (46)	32 (29)	0.005
Previous prostatectomy [n, (%)]	23 (8)	22 (11)	1 (1)	0.001
Comorbidity [n, (%)]	266 (86)	183 (92)	83 (76)	< 0.001
Cardiovascular disease [n, (%)]	230 (75)	156 (79)	74 (67)	0.029
Chronic obstructive pulmonary disease [n, (%)]	57 (19)	45 (23)	12 (11)	0.014
Chronic nephropathy [n, (%)]	41 (13)	28 (14)	13 (12)	0.604
Liver cirrhosis [n, (%)]	9 (3)	6 (3)	3 (3)	1.000
Diabetes [n, (%)]	78 (25)	58 (29)	20 (18)	0.040
Active smoking [n, (%)]	42 (14)	28 (14)	14 (13)	0.863
Anticoagulant treatment [n, (%)]	69 (22)	49 (25)	20 (18)	0.202
Comorbidity more than one [n, (%)]	176 (57)	129 (65)	47 (43)	< 0.001
Hernia type [n, (%)] Lateral Medial Femoral	227 (74) 67 (22) 14 (4)	156 (79) 35 (18) 7 (3)	71 (65) 32 (29) 7 (6)	0.023
Bilateral hernia repair [n, (%)]	77 (25)	38 (19)	39 (36)	0.002
Unilateral hernia repair [n, (%)] Right Left	137 (59) 94 (41)	96 (60) 64 (40)	41 (58) 30 (42)	0.748
Inguinoescrotal hernia [n, (%)]	98 (32)	70 (35)	28 (26)	0.097
Recurrent hernia [n, (%)]	38 (12)	27 (14)	11 (10)	0.374
Operative time (min) [mean (SD)]	111.5 (48)	98.5 (46.2)	135.1 (42.1)	< 0.001
Intraoperative complications [n, (%)]	3 (1)	2 (1)	1 (1)	0.709
Postoperative complication [n (%)]	104 (34)	73 (37)	31 (28)	0.133
Clavien Dindo classification of postoperative complications [n, (%)] None I II III IV V	204 (66) 89 (29) 12 (4) 2 (0.7) 0 (0) 1 (0.3)	125 (63) 58 (29) 12 (6) 2 (1) 0 (0) 1 (1)	79 (72) 31 (28) 0 (0) 0 (0) 0 (0) 0 (0)	0.057
Hematoma [n, (%)]	27 (9)	21 (11)	6 (6)	0.126
Seroma [n, (%)]	72 (23)	47 (24)	25 (23)	0.841
Acute urinary retention [n, (%)]	5 (2)	4 (2)	1 (1)	0.658
Length of stay (days) [mean (SD)]	1.4 (2.3)	1.7 (2.9)	1 (0.2)	0.021
Hernia recurrence [n, (%)]	6 (2)	2 (1)	4 (4)	0.192
Chronic postoperative inguinal pain [n, (%)]	8 (3)	4 (2)	2 (4)	0.463

*ASA* American Society of Anesthesiologists, *BMI* body mass index

### Hernia characteristics

In total, 77 patients (25%) underwent bilateral inguinal hernia surgery. Most patients with bilateral inguinal hernias were repaired via the R-TAPP approach (19 vs. 36%, *P* = *0.002*). In the open preperitoneal approach group, there was a larger proportion of lateral hernias, while in the R-TAPP group, medial hernias were more common (*P* = *0.023*). There were no differences between the groups in terms of repair of inguinoscrotal or recurrent hernias (Table 1).

### Operative and postoperative outcomes after 90 days

In the open preperitoneal approach group, 30 patients (15%) underwent surgery under spinal anesthesia, while the rest underwent surgery under general anesthesia. The R-TAPP procedure was significantly longer than the open preperitoneal approach (135.1 ± 42.1 min vs. 98.5 ± 46.2 min, *P* < *0.001*). In the R-TAPP repair group, one patient needed conversion to open surgery due to the presence of dense intra-abdominal adhesions.

The overall rate of postoperative complications at 90 days was 34% (n = 134), with no significant differences between the groups. The most common postoperative complications were seroma (n = 73; 23%), hematoma (n = 27; 9%) and acute urinary retention (n = 5; 2%), with no significant differences between the groups. The readmission rate related to inguinal hernia repair was 1.6% (n = 5) in the total series. Compared to the open preperitoneal group, the R-TAPP group had no report of readmission, and the difference was not significant (*P* = *0.164*). According to our multivariate analysis, the independent variables significantly associated with postoperative complications at 90 days were ASA grade $\ge$ III (OR, 1.763; 95% CI, 1.068–3.994; *P* = *0.027*) and inguinoscrotal hernia (OR, 2.371; 95% CI, 1.407–3.994; *P* = *0.001*). The length of hospital stay was significantly shorter in the R-TAPP group than in the open preperitoneal group (open preperitoneal mean 1.7 days, SD 2.9 days vs. R-TAPP 1, SD 0.2 days, *P* = *0.021*). (Table 2).

**Table 2:** Univariable and multivariable analysis of complications

	Complications			
Variables	Univariable analysis		Multivariable analysis	
	OR (95% CI)	P value	OR (95% CI)	P value
Patient age (y) < 75 (n = 141) ≥ 75 (n = 167)	1 1.165 (0.724 – 1.875)	0.528		
Sex Male (n = 272) Female (n = 36)	3.548 (1.336 – 9.419) 1	0.011	0.430 (0.157 – 1.181) 1	0.102
BMI < 30 (n = 277) ≥ 30 (n = 31)	1 1.980 (0.937 – 4.185)	0.073		
ASA score I/II (n = 169) III/IV (n = 139)	1 1.804 (1.120 – 2.907)	0.015	1 1.763 (1.068 – 2.908)	0.027
Previous abdominal surgery Yes (n = 122) No (n = 186)	0.988 (0.610 – 1.602) 1	0.962		
Comorbidity Yes (n = 266) No (n = 42)	0.905 (0.458 – 1.788) 1	0.774		
Cardiovascular disease Yes (n = 230) No (n = 78)	1.200 (0.691 – 2.084) 1	0.517		
Chronic Obstructive Pulmonary disease Yes (n = 57) No (n = 251)	0.801 (0.429 – 1.495) 1	0.486		
Chronic nephropathy Yes (n = 41) No (n = 267)	1.154 (0.582 – 2.288) 1	0.682		
Liver cirrhosis Yes (n = 9) No (n = 299)	1.592 (0.418 – 6.059) 1	0.495		
Diabetes Yes (n = 78) No (n = 230)	0.902 (0.521 – 1.559) 1	0.711		
Anticoagulant treatment Yes (n = 69) No (n = 239)	1.465 (0.843 – 2.544) 1	0.176		
Active smocking Yes (n = 42) No (n = 266)	1.570 (0.809 – 3.046) 1	0.182		
Comorbidity more than one Yes (n = 104) No (n = 204)	1.237 (0.765 – 2.001) 1	0.385		
Inguinoscrotal hernia Yes (n = 98) No (n = 210)	2.640 (1.601 – 4.354) 1	< 0.001	2.371 (1.407 – 3.994) 1	0.001
Bilateral hernia Yes (n = 104) No (n = 204)	1.257 (0.735 – 2.150) 1	0.404		
Recurrent hernia Yes (n = 38) No (n = 270)	0.572 (0.260 -1.258) 1	0.165		
Type of approach Open preperitoneal (n = 198) R-TAPP (n = 110)	1 0.672 (0.405 – 1.114)	0.123	1 0.856 (0.501 – 1.463)	0.570

### Long-term outcomes

The median follow-up period was 15 months (IQR: 7–26). The hernia recurrence rate in the entire cohort was 2% (n = 6). There were no significant differences between patients who underwent open preperitoneal repair and those who underwent robotic-assisted repair. (*P* = *0.192*) (Table 1). In the R-TAPP repair group, 2 patients developed an incisional hernia at the umbilical trocar and required subsequent mesh repair.

The CPIP rate in the entire series was 3% (n = 8) and was similar in both groups (*P* = *0.463*). No patients in the open preperitoneal approach group presented moderate or severe pain, while in the R-TAPP group, one patient presented moderate pain and another presented severe pain, although these differences were not significant (P = 0.178) (Table 1).

## Discussion

In the present study, which compared two posterior approaches for inguinal hernia repair, open and laparoscopic robot-assisted, both techniques presented similar short- and long-term postoperative results. The robot-assisted approach was a significantly longer procedure than the open preperitoneal approach, but the latter required a longer hospital stay. An ASA score ≥ III and inguinoscrotal hernia were found to be independent risk factors associated with 90-day postoperative complications.

Until the most recent update of the HerniaSurge clinical guidelines, the Lichtenstein procedure and minimally invasive techniques were recommended as the best evidence-based options for inguinal hernia repair, especially when performed by experts [2]. According to these guidelines, the open preperitoneal approach was not considered an effective alternative due to the limited evidence available concerning its use [2]. However, the latest update suggests that the open preperitoneal approach is at least comparable or even favorable with respect to the Lichtenstein technique in terms of recurrence rate, postoperative pain rate, and recovery time and is equivalent to laparo-endoscopic techniques [19]. The postoperative results of robot-assisted repair are similar to those of open surgery, with patients undergoing robotic surgery consuming less analgesics [20]. However, most of these studies included open anterior repairs as comparators; moreover, not using the same repair location as mesh placement is associated with greater postoperative pain and longer recovery time [19]. In our study, the robot-assisted approach and the open preperitoneal technique presented equivalent results in terms of postoperative morbidity, CPIP, and recurrence rates. In a previous study comparing robotic-assisted repair with the open preperitoneal TREPP technique, the authors reported equivalent results with both techniques in terms of recurrence rates and quality of life [9]. However, few studies have compared these two approaches. This is likely because open preperitoneal access is not performed routinely, and many surgeons are unaware of the advantages of this approach [3]. In the present study, the procedures were performed by high-volume surgeons who had become proficient in both techniques, which allows more homogeneous results and allows us to reinforce the importance of experience and training in abdominal wall surgery instead of only the choice of surgical technique [21].

In our study, postoperative complication rates after open and robotic-assisted preperitoneal hernia repairs were similar in terms of seroma formation, hematoma, and acute urinary retention, even though the patients in the open surgery group were older and had greater comorbidities. Allowing repair via access to the posterior wall of the inguinal region with the placement of a mesh in the preperitoneal space in patients with significant comorbidities and multiple previous abdominal interventions is one of the advantages of the open preperitoneal approach.

The R-TAPP procedure was longer, as has been reported in previous studies [7, 9]. However, patients in this group had shorter hospital stays than those in the open group had; however, these findings should be interpreted with caution given that the difference was only one day, and its clinical relevance is questionable.

No differences in postoperative chronic inguinal pain were observed between the groups, which is to be expected given that both procedures avoid the dissection and manipulation of the nerves in the inguinal canal and that improper fixations are associated with a higher risk of chronic pain [22]. There were also no differences in terms of hernia recurrence, which is probably because both techniques allow complete visualization of the myopectineal orifice and mesh placement with sufficient overlap at that level. Overall, training on the open preperitoneal approach should be improved and more frequent in current surgical practice because the approach is excellent for inguinal hernia repair and can be as effective as the robotic-assisted laparoscopic approach because of the advantages mentioned above. Notably, 2% of hernias developed at trocar sites in our R-TAPP series. There are few studies that have reported this complication; in a series of 429 patients, 0.5% of hernias developed at the trocar site at the umbilical level [23]. Factors such as the choice of anatomical trocar placement and tissue manipulation during robotic-assisted procedures deserve thorough exploration to reduce the risk of this complication [24].

Our multivariate analysis indicated that an ASA grade ≥ III and inguinoscrotal hernia were risk factors for postoperative complications. Several authors have reported that the presence of associated comorbidities, determined by the American Society of Anesthesiologists (ASA) score, is a risk factor for morbidity after inguinal hernia repair [25]. On the other hand, inguinoscrotal hernias have been associated with a higher risk of complications, regardless of the repair method used [26]. In this sense, we consider it important to follow the recommendations in the clinical guidelines because patients with high comorbidity and those with inguinoscrotal hernias should be operated on in tertiary hospitals with teams that include surgeons experienced in surgeries involving the abdominal wall [2, 27].

This study is limited by its retrospective nature, single-center design, selection bias and absence of data on quality of life and costs. Furthermore, the surgeons’ learning curve could potentially influence the results of the robotic approach. However, our results may be interesting in terms of external validity, as the patients were treated in a specific abdominal wall surgery unit with surgeons experienced in both techniques, excluding the variations in the confounding variables that arise from including patients treated in different centers. Furthermore, comparing open preperitoneal techniques with robot-assisted techniques is valid and useful because both focus on the same anatomical plane and seek to achieve the best results; moreover, very few studies have compared these methods.

A question that arises is which surgical technique should be selected. From the perspective of our unit (specialized in abdominal wall surgery) and based on the current data, it appears that both techniques are equally effective in terms of short- and long-term postoperative outcomes, suggesting that the choice between them could be influenced by factors that extend beyond clinical efficacy. These factors can vary between hospitals and geographically and may depend on the following factors: 1. Surgeon preference—the surgeon may select the technique they are most experienced in and most comfortable with performing; 2. Hospital resources—the robotic equipment must be available and accessible and the hospital must have the capacity meet the financial demands associated with such equipment, as robotic procedures are more time consuming and expensive; 3. Recovery and discharge times—the preference for a shorter hospital stay may lead to the technique that is known to have a quicker recovery and earlier discharge; 4. Logistical factors—including scheduling of surgery, operating room time availability, and anxiety associated with having a waiting list can also be important considerations; 5. Patient preferences—such as recovery time, incision size, and the perception of advanced technology can also influence patient preference.

In summary, this study indicated that both open preperitoneal and robotic-assisted laparoscopic approaches have comparable outcomes in terms of postoperative complication, chronic pain, and hernia recurrence rates when performed by experienced surgeons trained in both techniques. The open preperitoneal approach is beneficial because the mesh is placed in the preperitoneal space in patients with high comorbidity, thus shortening the operative time. Finally, based on our results, the choice of the most appropriate treatment should be based on the patient’s individual factors, the surgeon’s experience, and the resources available in the healthcare system.

## Data Availability

Not applicable.
